# Gene deletion of γ‐actin impairs insulin‐stimulated skeletal muscle glucose uptake in growing mice but not in mature adult mice

**DOI:** 10.14814/phy2.15183

**Published:** 2022-02-27

**Authors:** Jonas R. Knudsen, Agnete B. Madsen, Zhencheng Li, Nicoline R. Andersen, Peter Schjerling, Thomas E. Jensen

**Affiliations:** ^1^ Section for Molecular Physiology Department of Nutrition, Exercise and Sports University of Copenhagen Copenhagen Denmark; ^2^ Department of Orthopedic Surgery M Institute of Sports Medicine Copenhagen Bispebjerg Hospital Copenhagen Denmark

**Keywords:** γ‐actin, glucose uptake, skeletal muscle

## Abstract

The cortical cytoskeleton, consisting of the cytoplasmic actin isoforms β and/or γ‐actin, has been implicated in insulin‐stimulated GLUT4 translocation and glucose uptake in muscle and adipose cell culture. Furthermore, transgenic inhibition of multiple actin‐regulating proteins in muscle inhibits insulin‐stimulated muscle glucose uptake. The current study tested if γ‐actin was required for insulin‐stimulated glucose uptake in mouse skeletal muscle. Based on our previously reported age‐dependent phenotype in muscle‐specific β‐actin gene deletion (^−/−^) mice, we included cohorts of growing 8–14 weeks old and mature 18–32 weeks old muscle‐specific γ‐actin^−/−^ mice or wild‐type littermates. In growing mice, insulin significantly increased the glucose uptake in slow‐twitch oxidative soleus and fast‐twitch glycolytic EDL muscles from wild‐type mice, but not γ‐actin^−/−^. In relative values, the maximal insulin‐stimulated glucose uptake was reduced by ~50% in soleus and by ~70% in EDL muscles from growing γ‐actin^−/−^ mice compared to growing wild‐type mice. In contrast, the insulin‐stimulated glucose uptake responses in mature adult γ‐actin^−/−^ soleus and EDL muscles were indistinguishable from the responses in wild‐type muscles. Mature adult insulin‐stimulated phosphorylations on Akt, p70S6K, and ULK1 were not significantly affected by genotype. Hence, insulin‐stimulated muscle glucose uptake shows an age‐dependent impairment in young growing but not in fully grown γ‐actin^−/−^ mice, bearing phenotypic resemblance to β‐actin^−/−^ mice. Overall, γ‐actin does not appear required for insulin‐stimulated muscle glucose uptake in adulthood. Furthermore, our data emphasize the need to consider the rapid growth of young mice as a potential confounder in transgenic mouse phenotyping studies.

## Graphical abstract

Cytoplasmic gamma actin may regulate insulin‐stimulated GLUT4 translocation and thereby glucose uptake. Muscle‐specific gamma actin gene deletion does not reduce insulin‐stimulated muscle glucose uptake in adult mice. This suggests that gamma‐actin is not required for insulin‐stimulated muscle glucose uptake.

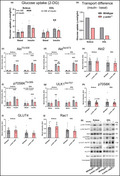

**New findings**

1. What is the central question of this study?

Is γ‐actin required for insulin‐stimulated glucose uptake in mouse skeletal muscle fibers.

2. What is the main finding and its importance?

Reduced insulin‐stimulated glucose uptake was observed in young, growing but not mature adult mice, suggesting a growth x genotype interaction.

Overall, however, γ‐actin is not required for insulin‐stimulated glucose uptake in adult mouse muscle.

This improves our mechanistic understanding of insulin‐regulated metabolism in adult muscle and emphasizes the need to consider the rapid growth of young mice as a potential confounder in transgenic mouse phenotyping studies.

## 1 INTRODUCTION

Whole‐body insulin‐resistance has been proposed to accelerate a range of non‐communicable diseases (Czech, 2020; DeFronzo et al., 2015; González‐Muniesa et al., 2017; Scully et al., 2021). Skeletal muscle is quantitatively the largest site of post‐prandial insulin‐stimulated glucose disposal and a major site of insulin‐resistance (DeFronzo & Tripathy, 2009). Mechanistic understanding of how insulin increases glucose uptake into muscle fibers may therefore help prevent/treat muscle insulin‐resistance.

Insulin and other stimuli such as contraction increase skeletal muscle glucose uptake (MGU) by translocating glucose transporter 4 (GLUT4) from intracellular stores to the cell‐surface (Jensen et al., 2014; Sylow et al., 2017, 2021). A number of studies in immortalized skeletal muscle and adipose cell culture have suggested that an insulin‐stimulated dynamic remodelling of the cortical actin cytoskeleton, mainly consisting of β‐actin and/or γ‐actin isoforms (Perrin & Ervasti, 2010; Tondeleir et al., 2009; Vedula & Kashina, 2018), is involved in GLUT4 translocation (Klip et al., 2019; Sylow et al., 2021). Notably, the actin remodelling process appears to become insulin‐resistant in cultured myotubes exposed to high glucose and insulin (Tong et al., 2001). The findings in cultured cells are supported by several studies in rodents. Hence, acute pharmacological depolymerisation of the actin cytoskeleton inhibits insulin‐stimulated GLUT4 translocation and glucose uptake in incubated rat and mouse muscles (Brozinick et al., 2004; Sylow et al., 2013a,b; Sylow et al., 2015). Furthermore, disruption of a number of actin‐regulating proteins reduces insulin‐stimulated GLUT4 translocation and glucose uptake (González‐Jamett et al., 2017; Kee et al., 2015; Masson et al., 2020; Sylow et al., 2013a; Toyoda et al., 2011; Ueda et al., 2010; Vahsen et al., 2006; Wang et al., 2011; Zong et al., 2009). Finally, high‐fat diet induced insulin resistance in rodents correlated with decreased F‐actin immunofluorescent staining (Grice et al., 2019; Habegger et al., 2012).

We previously used conditional muscle‐specific β‐actin^−/−^ mice to investigate if the cytoplasmic β‐actin isoform was required for MGU stimulation by different stimuli, including insulin (Madsen et al., 2018). Surprisingly, no genotype‐differences in glucose uptake‐responsiveness were observed, except for a reduction in maximal insulin‐stimulated glucose uptake in 8–13 weeks old mice. This difference was absent in 18–23 weeks old mice, suggesting this to be an indirect and transient growth‐dependent phenotype. A similar growth‐dependent phenotype has been reported for other skeletal muscle endpoints such as satellite cell‐dependent hypertrophy (Murach et al., 2017).

One possibility to explain the normal MGU in mature adult mouse muscles lacking cytoplasmic β‐actin might be that cytoplasmic γ‐actin is the predominant isoform regulating insulin‐stimulated GLUT4 translocation and MGU, consistent with the stronger progressive myopathy phenotype of γ‐actin^−/−^ (Sonnemann et al., 2006) compared to β‐actin^−/−^ mice (Prins et al., 2011). To test this, we analysed maximal insulin‐stimulated glucose uptake and cell signaling in incubated soleus and extensor digitorum longus (EDL) muscles from young growing 9–14 weeks old and mature adult 18–32 weeks old cohorts of γ‐actin^−/−^ mice and corresponding littermates.

## 2 METHODS AND MATERIALS

### 2.1 Animals

#### 2.1.1 Ethical approval

All experiments and breeding protocol were approved by the Danish Animal Experiments Inspectorate (license: 2017‐15‐0201‐01311) and carried out in accordance with the European Convention for the Protection of Vertebrate Animals used for Experimental and Other Scientific Purposes. The experiments conformed to the principles and regulations as described in the editorial by Grundy (Grundy, 2015).

The conditional γ‐actin^−/−^ mice were littermates from the cross‐breeding of hemizygous human α‐skeletal actin (HSA)‐Cre and γ‐actin^flox/flox^ mice originally generated by prof. James M Ervasti, University of Minnesota, USA (Sonnemann et al., 2006). In brief, the γ‐actin^−/−^ mice develop a progressive myopathy with muscle weakness, centronucleated fibers and more variable fiber sizes being apparent at 3 months mainly in different type II‐fiber dominated muscles but not soleus muscle (Sonnemann et al., 2006). Due to poor breeding performance of the mouse strain and no discernible sex‐dependent differences in measured endpoints, the male and female mouse data were pooled for statistical analyses. Wild‐type and γ‐actin^−/−^ mice were littermates. Mice were maintained at 22–24°C on a 12‐h light/dark cycle with *ad libitum* access to water and rodent chow diet. Mice were categorized as growing when aged 9–14 weeks and mature adults when aged 18–32 weeks.

### 2.2 Genotyping

Muscle pieces (5–10 mg) were lysed overnight at 55°C in 100 µl of DirectPCR Lysis Reagent (Tail) (250‐101‐T, Nordic BioSite) with freshly added 0.2 mg/ml proteinase K (Roche Diagnostics). The lysate was centrifuged at 1250 g for 5 min at room temperature, and the supernatant was kept for DNA extraction. The released DNA was diluted ten‐fold in dilution buffer (10 mM Tris, 1 mM EDTA, pH 8.0), and 5 μl of this dilution was then amplified in a 25‐μl SYBR Green polymerase chain reaction (PCR) containing 1× Quantitect SYBR Green Master Mix (Qiagen) and 200 nM of each primer (gDNA: TCCAGCACGTGGGTCTTAGAGG and CTGGCAGGTAGGCTCAGCAGGT, γ‐actin flox: GGGCGTGACGAGTCATTTTGTG and GAATCGATGCC CCTGAATTCATAAC). The amplification was monitored in real time using the MX3005P real‐time PCR machine (Stratagene). All reactions were performed in triplicate, and the mean C_T_ was used for calculations. Flox abundance was calculated as 2^(C_T Flox_ − C_T gDNA_) and normalized to the geometric average of all mice.

### 2.3 Muscle incubation and *ex vivo* 2‐DG uptake

The mice were anaesthetized (pentobarbital 6 mg and 0.2 mg lidocaine/100 g body weight, intraperitoneal injection), followed by excision of soleus and EDL muscles. Immediately after removal of the muscles the mice were killed by cervical dislocation. The excised muscles were allowed to rest for 30 min in incubation chambers (Multi Myograph System, Danish Myo‐Technology) containing 30°C Krebs–Ringer–Henseleit (KRH) buffer with 8 mM mannitol and 2 mM pyruvate as previously described (Knudsen et al., 2020). For insulin stimulation, 30 min rested muscles were stimulated with 60 nM insulin for 20 min. 2‐Deoxyglucose (2‐DG) transport was measured during the last 10 min of incubation using [^3^H]‐2‐Deoxy‐D‐glucose ([^3^H]‐2‐DG) and [^14^C]‐mannitol radioactive tracers as previously described (Knudsen et al., 2020). Immediately after *ex vivo* incubation, the muscles were washed in ice‐cold KRH buffer and snap‐frozen in liquid nitrogen.

### 2.4 Immunoblotting

Tissue was homogenized in ice‐cold lysis buffer (0.05 M Tris base pH 7.4, 0.15 M NaCl, 1 mM EDTA and EGTA, 0.05 M sodium fluoride, 5 mM sodium pyrophosphate, 2 mM sodium orthovanadate, 1 mM benzamidine, 0.5% protease inhibitor cocktail (P8340; Sigma Aldrich), and (1% NP‐40) using a TissueLyser II bead‐mill (QIAGEN, Hilden, Germany). After rotating the homogenates end‐over‐end at 4°C for 30 min, supernatants were collected by centrifugation (18,327×g) for 20 min at 4°C. Protein concentrations were determined using Pierce™ BCA Protein Assay Kit (23225; Thermo Fisher‐Scientific). Lysates diluted to equal protein concentration and containing Laemmlii sample buffer (62.5 mM Tris [pH 6.8], 2% SDS, 10% glycerol, 0.1 M DTT, 0.01% bromophenol blue) were heated at 95°C for 5 min prior to storage or gel loading.

Equivalent amounts of protein were separated by SDS‐PAGE gels and semi‐dry transferred to PVDF membranes (Immobilon^®^‐P Transfer Membranes, Millipore). The membranes were first blocked in Tris buffered saline with 0.05% Tween 20 (TBS‐T) containing either 3% skimmed milk powder or BSA for 30 min at room temperature, followed by overnight incubation with the indicated primary antibodies at 4°C. The following primary antibodies were used: p‐p70S6K Thr389 (CST, #9205), p70S6K (CST, #9202), p‐Akt Thr308 (CST, #9295), p‐Akt Ser473 (CST, #9271), Akt (CST, #9272), p‐ULK1 Ser757 (CST, #6888), Rac1 (BD Biosciences, 61065), GLUT4 (Thermo Fisher, PA1‐1065). On the next day, membranes were incubated with the corresponding horseradish peroxidase‐conjugated secondary antibody (#111‐035‐045, #115‐035‐062, #113‐035‐147; Jackson ImmunoResearch) for 1 h at room temperature, washed in TBS‐T, and then visualized using the enhanced chemiluminescence (Forte Western HRP Substrate, Millipore) and ChemiDocTM MP Imaging System (Bio‐Rad). Individual band intensities were quantified and normalized to the average intensity of the full data set. To verify even transfer and similar total protein loading, membranes were washed with TBS‐T and stained with Coomassie Brilliant Blue.

### 2.5 Data presentation and statistics

Results are presented as means with individual data points. Statistical testing was performed using unpaired two‐tailed *t*‐test or two‐way analysis of variance (ANOVA) with or without repeated measures as described in the figure legends. If significant (*p* < 0.05) ANOVA main effects or interactions were found, Tukey's post hoc test was used for multiple comparisons, unless otherwise noted. Non‐significant *p* values between 0.5 and 0.1 are shown on the figures. The statistical analyses were carried using GraphPad Prism Vers. 9.

## 3 RESULTS

**FIGURE 1:**
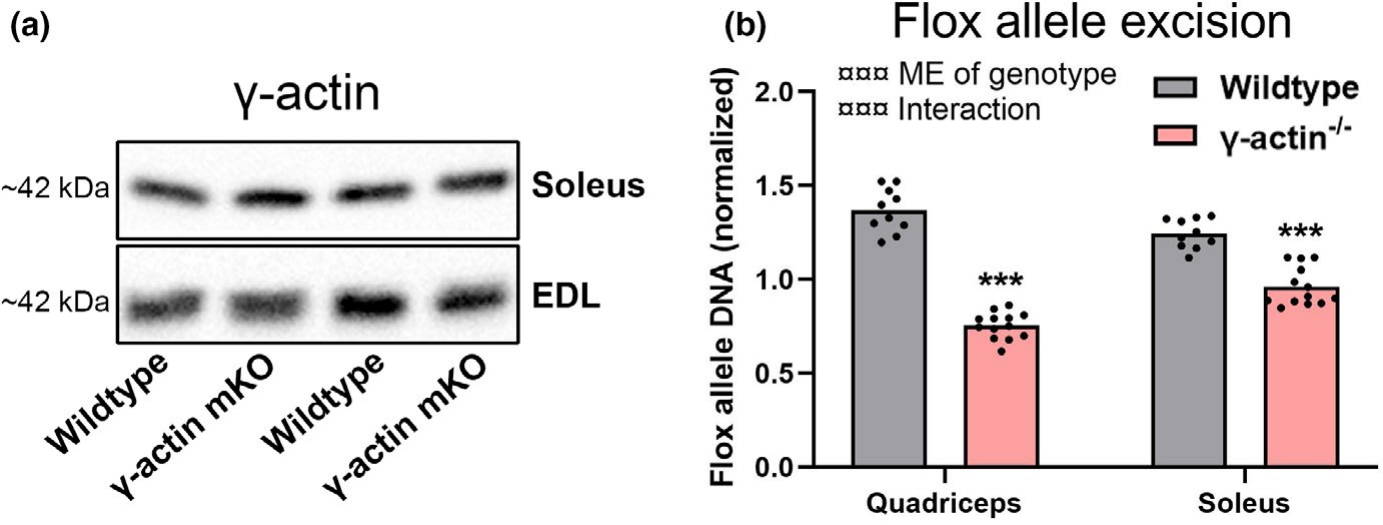
Verification of the γ‐actin^−/−^ genotype. (a) Immunoblotting against γ‐actin in isolated soleus and EDL muscles from wild‐type and muscle specific γ‐actin^−/−^ mice. (b) Quantification of the flox allele in quadriceps and soleus muscles of wild‐type and γ‐actin^−/−^ mice. Two‐way repeated measures ANOVA was performed followed by a Sidak's post hoc test. ¤¤¤*p* < 0.001 ANOVA effects. ME = main effect. ***p < 0.001 effect of genotype. For wild‐type and γ‐actin^−/−^ *n* = 10 and 13 animals respectively

To validate muscle specific removal of γ‐actin we first immunoblotted whole‐muscle lysate using a γ‐actin antibody. However, we found similar γ‐actin expression in the wild‐type and ^−/−^ lysate (Figure 1a), likely due to γ‐actin from other cell types dominating γ‐actin pool in whole‐muscle lysate. Thus, we next verified the effective excision of the floxed γ‐actin gene in myofibers by PCR similar to our previous studies (Li et al., 2021; Madsen et al., 2018). This yielded a ~50% reduction in floxed gene content in quadriceps muscle and ~30% in soleus (Figure 1b). A reduction of this magnitude is in agreement with the proportion of myofiber nuclei to total nuclei in skeletal muscle (Wang et al., 2014), and supports that efficient muscle‐specific excision of the γ‐actin gene had occurred as intended.

**FIGURE 2:**
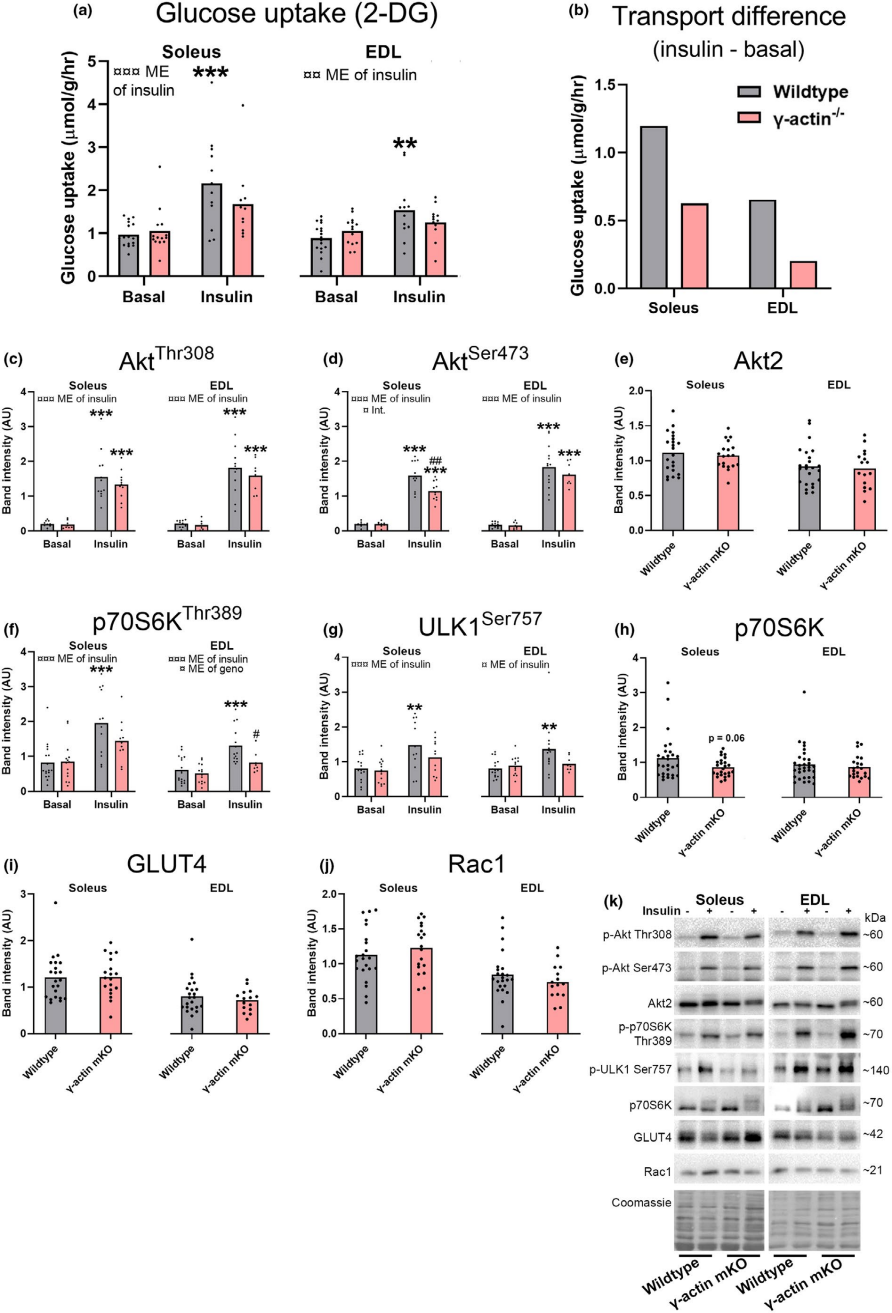
Impaired insulin‐stimulated glucose uptake in soleus and EDL muscles from growing muscle‐specific γ‐actin^−/−^ mice. Isolated soleus and EDL muscles excised from growing (8–14 weeks old) wild‐type and muscle specific γ‐actin^−/−^ mice were incubated in constantly oxygenated Krebs–Ringer–Henseleit buffer at 30°C for 30 min and then stimulated with insulin (60 nM) or kept in the unstimulated state for 10 min before a final 10 min of [^3^H]‐2‐Deoxy‐D‐glucose (2‐DG) tracer accumulation. (a) glucose transport into soleus and EDL muscles. (b) insulin‐stimulated muscle glucose uptake calculated as the difference between insulin and basal‐stimulated transport. (c) phospho(p)‐Akt Ser473, (d) p‐Akt Thr308, (e) Akt2, (f) p‐p70S6K Thr389, (g) p‐ULK1 Ser757, (h) p70S6K, (i) GLUT4 and (j) Rac1 levels quantified from soleus and EDL. (k) representative blots of the quantified proteins in (c–j). Ser = serine, Thr = threonine, ME = main effect, Int. = interaction, geno. = genotype, ins. = insulin. For (a, c, d, f and g) two‐way ANOVA was performed followed by a Tukey's post hoc test in case of ANOVA effect. For (e, h, i, and j), a *t* test was performed. ¤/¤¤/¤¤¤*p* < 0.1/0.01/0.001 ANOVA effect. */**/***p < 0.05/0.01/0.001 effect of insulin, #*p* < 0.05 different from corresponding wild‐type group. For soleus wild‐type basal, γ‐actin^−/−^ basal, wild‐type insulin and γ‐actin^−/−^ insulin *n* = 17, 14, 12, 11 respectively and for EDL in the same order *n* = 17, 14, 12, 12

In growing wild‐type mice 9 to 14 weeks of age insulin increased the muscle glucose uptake by ~125% in soleus and ~75% in EDL (Figure 2a). In contrast, although the insulin‐stimulated muscles on average were ~60% and ~20% higher than basal control soleus and EDL muscles from the γ‐actin^−/−^ mice, the effect of insulin on MGU was neither significant in soleus (*p* = 0.13) nor in EDL (*p* = 0.67) (Figure 2a). The observed difference (insulin – corresponding basal MGU) were ~50% lower in soleus and ~70% lower in EDL from γ‐actin^−/−^ compared to wild‐type (Figure 2b). As expected insulin increased phospho‐Akt at Thr308 and Ser473 in wild‐type and γ‐actin^−/−^ mice in both soleus and EDL muscles (Figure 2c and d). However, γ‐actin^−/−^ mice had lower insulin‐stimulated phospho‐Akt levels at Ser473 in soleus than the wild‐type mice (Figure 2d). Total Akt2 protein expression was similar between groups (Figure 2e). This prompted us to investigate Akt‐mediated mTORC1‐regulated phosphorylation sites in the γ‐actin^−/−^ mice. Interestingly, only the wild‐type mice responded to insulin at phospho‐p70S6K Thr389 and phospho‐ULK1 Ser757 (Figure 2f and g) and particularly the insulin‐stimulated phospho‐p70S6K Thr389 were lower in the EDL muscles from γ‐actin^−/−^ mice when compared to the insulin‐stimulated wild‐type muscles (Figure 2f). Total p70S6K expression was similar across groups (Figure 2h). Total GLUT4 and Rac1 protein expression were not different between the genotypes in any of the muscles (Figure 2i and j). Representative western blots are presented in Figure 2k. In conclusion, insulin‐stimulated MGU and cell signaling readout are less responsive in growing γ‐actin^−/−^ mice compared to wild‐type.

**FIGURE 3:**
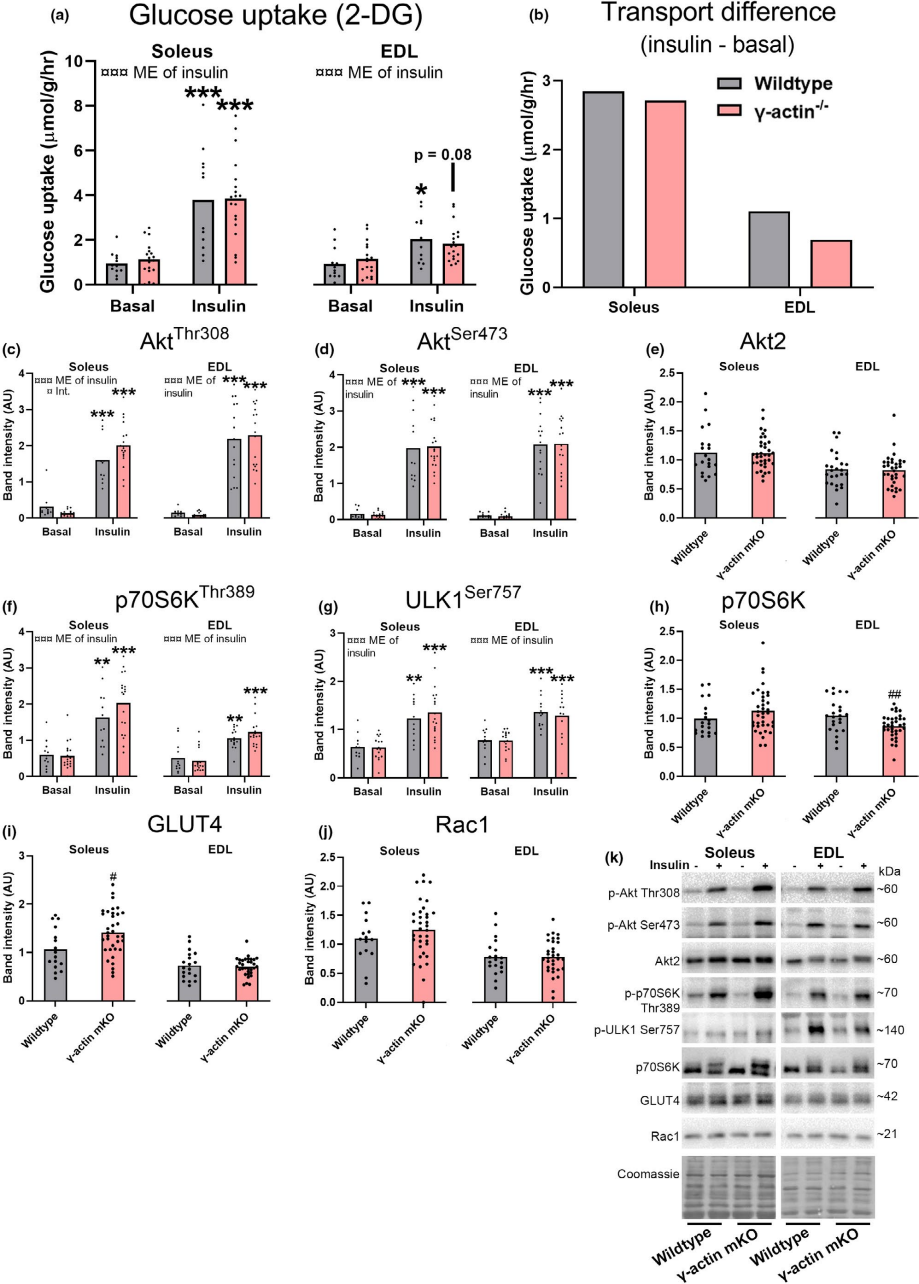
Similar insulin‐induced glucose uptake in soleus and EDL muscles from adult wild‐type and muscle‐specific γ‐actin^−/−^ mice. Isolated soleus and EDL muscles excised from adult (18–32 weeks old) wild‐type and muscle specific γ‐actin^−/−^ mice were incubated in constantly oxygenated Krebs–Ringer–Henseleit buffer at 30°C for 30 min and then stimulated with insulin (60 nM) or kept in the unstimulated state for 10 min before a final 10 min of [^3^H]‐2‐Deoxy‐D‐glucose (2‐DG) tracer accumulation. (a) glucose transport into soleus and EDL muscles. (b) insulin‐stimulated muscle glucose uptake calculated as the difference between insulin and basal‐stimulated transport. (c) phospho(p)‐Akt Ser473, (d) p‐Akt Thr308, (e) Akt2, (f) p‐70S6K Thr389, (g) p‐ULK1 Ser757, (h) p70S6K, (i) GLUT4 and (j) Rac1 levels quantified from soleus and EDL. (k) Representative blots of the quantified proteins in (c–j). Ser = serine, Thr = threonine, ME = main effect, Int. = interaction, geno. = genotype, ins. = insulin. For (a, c, d, f and g) two‐way ANOVA was performed followed by a Tukey's post hoc test in case of ANOVA effect. For (e, h, i and j) a *t* test was performed. ¤/¤¤¤*p* < 0.1/0.001 ANOVA effect. **/***p < 0.01/0.001 effect of insulin, ##*p* < 0.01 different from corresponding wild‐type group. For soleus wild‐type basal, γ‐actin^−/−^ basal, wild‐type insulin and γ‐actin^−/−^ insulin *n* = 12, 18, 12, 19 respectively and for EDL in the same order *n* = 12, 17, 12, 19

In mature adult 18–32 week old mice, insulin increased MGU in soleus muscles from both wild‐type and γ‐actin^−/−^ mice (Figure 3a). Insulin also increased MGU in wild‐type EDL muscles and tended to increase MGU in γ‐actin^−/−^ muscles (*p* = 0.08). Specifically, insulin increased the MGU by ~300% in wild‐type soleus and ~240% in soleus from γ‐actin^−/−^ mice and for EDL the increases were ~120% and ~60% for wild‐type and γ‐actin^−/−^, respectively (Figure 3a). The calculated effect of insulin on MGU in soleus and EDL is shown in Figure 3b. Signaling‐wise, insulin increased phospho‐Akt Thr308 and Ser473 in both muscles irrespective of genotype, and with no difference in total Akt2 protein content (Figure 3c–e). Insulin also increased phospho‐p70S6K Thr389 and phospho‐ULK1 Ser757 irrespective of genotype (Figure 3f and g). Interestingly, total p70S6K protein content was lower in EDL muscles from γ‐actin^−/−^ mice than in corresponding wild‐type muscles (Figure 3h), while GLUT4 protein content was higher in soleus muscles from γ‐actin^−/−^ mice than in corresponding wild‐type muscles (Figure 3i). Rac1 protein content was not significantly affected by the genotype (Figure 3j). Representative western blots are presented in Figure 3k. In conclusion, insulin‐stimulated MGU and cell signaling in appear normal in mature‐adult γ‐actin^−/−^ mice although total protein content of GLUT4 and p70S6K may be altered by γ‐actin^−/−^ in soleus and EDL, respectively.

## 4 DISCUSSION

In the present study, we investigated whether γ‐actin rather than β‐actin might be required for insulin‐stimulated MGU. In young growing γ‐actin^−/−^ mice, the apparent ability of insulin to stimulate MGU in both soleus and EDL muscle was less compared to wild‐type since no significant effects of insulin were observed in the γ‐actin^−/−^ muscles. However, in mature adult mice, the effect of insulin was normal in the soleus γ‐actin^−/−^ muscles. Similarly, in EDL muscles there were no genotype differences on insulin‐stimulated MGU and the MGU was significantly increased in the wild‐type muscles and tended to increase in the γ‐actin^−/−^ muscles. This could indicate that the impaired insulin‐stimulated MGU observed in young γ‐actin^−/−^ mice is secondary to the previously reported impaired muscle growth and maturation (Sonnemann et al., 2006) and does not reflect a requirement for γ‐actin in the actual GLUT4 translocation/glucose uptake process.

Numerous pharmacological and transgenic rodent studies have previously suggested that insulin‐stimulated glucose uptake in adult skeletal muscle is dependent on the cortical actin‐cytoskeleton. Pharmacologically, this is evidenced by the marked reduction of insulin‐stimulated GLUT4 translocation and glucose uptake by the depolymerizing agent latrunculin B in ex vivo incubated rat epitrochlearis muscle (Brozinick et al., 2004) and of insulin, contraction and passive stretch‐stimulated glucose uptake in ex vivo incubated mouse soleus and EDL muscle (Sylow et al., 2013a,b; Sylow et al., 2015). In terms of transgenic mouse models, a number of KO models lacking proteins linked to actin regulation have been shown to display reduced insulin‐stimulated MGU and/or GLUT4 translocation. The proteins investigated in KO mice include 12/15‐Lipoxygenase (20 weeks old mice) (Vahsen et al., 2006), β1 integrin (14 weeks old mice) (Zong et al., 2009), Myo1c (8–10 weeks old mice) (Toyoda et al., 2011), Tropomyosin Tpm3.1 (12–16 weeks old mice) (Kee et al., 2015), dynamin‐2 (8–12 weeks old mice) (González‐Jamett et al., 2017), β‐catenin (drug‐inducible model, 10–20 weeks old mice at time of experiments) (Li et al., 2016), Rac1 (drug‐inducible model, 14–18 weeks at time of experiments (Sylow et al., 2013a,b; Sylow et al., 2015) Rac1 (constitutive, 8–10 weeks old mice) (Ueda et al., 2010) and PAK1 (16–24 weeks old mice) (Wang et al., 2011), although the PAK1 KO mouse phenotype does not seem robust (no difference observed in 10–24 weeks old mice) (Møller et al., 2020). In light of our age‐dependent phenotype, we specifically consider it possible that a genotype x growth interaction may have produced some of the observed insulin‐resistant phenotypes in the studies using young mice. Also, the reduced insulin‐stimulated glucose uptake could also reflect the involvement of these proteins in other processes than actin remodelling. For instance, although a role of Rac1‐stimulated actin remodelling is possible, Rac1 appears to regulate GLUT4 translocation and MGU during *in vivo* exercise via NOX2 mediated production of reactive oxygen species (Henríquez‐Olguin et al., 2019). Nonetheless, these studies as a whole indicate that the cortical actin cytoskeleton is involved in insulin‐stimulated GLUT4 translocation and glucose uptake in adult muscle similar to cultured cells.

The most obvious explanation of why neither β‐actin (Madsen et al., 2018) nor γ‐actin are required for insulin‐stimulated glucose uptake in mature adult muscle would be functional redundancy between the cytoplasmic β and γ isoforms. Cytoplasmic β and γ‐actin are 99% homologous and can produce heteromeric F‐actin in vitro (Bergeron et al., 2010; Müller et al., 2013). Muscle‐specific β and γ‐actin^−/−^ mice also develop similar phenotypes with gradually developing myopathies characterized by accelerated myofiber turnover and weakness (Prins et al., 2011; Sonnemann et al., 2006), which might also indicate overlapping functions. However, there is also evidence that β‐ and γ‐actin are part of distinct actin structures in different cell types (Perrin & Ervasti, 2010; Tondeleir et al., 2009; Vedula & Kashina, 2018), making the degree to which β‐ and γ‐actin work interchangeably *in vivo* a continuous point of debate. A study in muscle‐specific β/γ‐actin double ^−/−^ mice, preferably drug‐inducible in adult animals to minimize compensation during development, could be performed to directly test this. In contrast, cytoplasmic actins and muscle α‐actins, although also highly homologous (~93%), are generally thought to be non‐redundant in their functions (Perrin & Ervasti, 2010; Tondeleir et al., 2009; Vedula & Kashina, 2018), making skeletal muscle α‐actin less likely to compensate for the absence of γ‐actin. That said, ~2000x overexpression of γ‐actin in mouse muscle has also been shown to incorporate readily in skeletal muscle α‐actin filaments, although this cannot rescue the reduced life‐span of skeletal muscle α‐actin KO mice (Murach et al., 2017). Taken together, we speculate that β and γ‐actin in particular may be functionally redundant in the regulation of insulin‐stimulated MGU.

What might explain the smaller effect of insulin on MGU in young γ‐actin^−/−^ mice compared to wild‐type? Although the phosphorylation level of Akt Thr308 was similar between wild‐type and γ‐actin ^−/−^ mice, the gene‐deleted mice had lower insulin‐stimulated Akt Ser473 in soleus. This might reflect inhibition of insulin‐signaling secondary to inflammation and potentially other mechanisms which converge onto inhibition of IRS1 function (DeFronzo et al., 2015). A decreased structural integrity of γ‐actin^−/−^ muscle fibers might have exacerbated these mechanisms in young growing mice. However, increased muscle inflammation was not observed in γ‐actin^−/−^ mice although the specific age and muscle investigated was not reported (Sonnemann et al., 2006). Also, whether the relative subtle reduction in insulin‐signaling contributes to muscle insulin‐resistance is unclear due to the large spare capacity of Akt‐signaling to GLUT4 translocation (Hoehn et al., 2008). A related possibility might be increased production of reactive oxygen species (ROS). Hence, a ROS‐dependent loss of transverse microtubules has been described in β and γ‐actin^−/−^ muscles, partly depedendent on NOX2 (Nelson et al., 2020). Both NOX2 and other ROS sources such as mitochondria have been implicated in the development of insulin‐resistance, with considerable cross‐talk known to occur between the two (Henríquez‐Olguín et al., 2019). Microtubules are well‐described to serve as tracks for long‐range GLUT4 trafficking (Klip et al., 2019; Sylow et al., 2021). NOX2‐dependent ROS production is also known to increase with mechanical stress in cardiomyocytes and skeletal muscle (Pal et al., 2013; Prosser et al., 2011, 2013) and contractile activity in vitro and in vivo (Henríquez‐Olguin et al., 2019; Pal et al., 2013; Sakellariou et al., 2013). Based on this, increased ROS production due to mechanical hyperactivation of NOX2 or from other sources in growing γ‐actin^−/−^ muscle could contribute to the development of muscle insulin resistance.

Unfortunately, we were unable to directly demonstrate a reduction in γ‐actin in the muscles from the ^−/−^ mice using immunoblotting in the present study. This could indicate that the γ‐actin gene excission was ineffective in our ^−/−^ mice. However, since (a) the used model was previously validated as being a muscle‐fiber specific protein knockout mouse (Sonnemann et al., 2006) and (b) we observed the expected level of reduction in floxed gene content, we consider it more likely that γ‐actin from non‐muscle cell types dominated the γ‐actin signal in whole‐muscle lysate. Another study found that knocking out NOX4 specifically in endothelial cells caused a ~80% reduction in whole‐muscle NOX4 mRNA (Specht et al., 2021), clearly showing how other cell types than muscle fibers can dominate the whole‐muscle lysate signal. Thus, we believe that our used mice were γ‐actin^−/−^ although we acknowledge our inability to directly show this.

In summary, γ‐actin^−/−^ transiently impaired insulin‐stimulated MGU in young, growing mice but not mature adult mice. The impaired glucose uptake phenotype in young γ‐actin^−/−^ mice is thus likely an indirect effect provoked by rapid growth of the more fragile muscle fibers in γ‐actin^−/−^ mice. Since neither β‐actin nor γ‐actin gene deletion alone reduced insulin‐stimulated glucose uptake, we consider it likely that these actin isoforms are functionally redundant in adult skeletal muscle.

## CONFLICT OF INTEREST

Nothing to declare.

## AUTHOR CONTRIBUTION

T.E.J. conceived the study. All co‐authors performed experiments. J.R.K., A.B.M., and P.S. analyzed data. J.R.K. prepared figures. T.E.J. drafted the manuscript. All authors edited and revised the manuscript. All authors approved the final version of manuscript.
